# 1000 Animals Left Behind: Responder Experiences of the 2017 Edgecumbe Flood in New Zealand

**DOI:** 10.3390/ani14142083

**Published:** 2024-07-17

**Authors:** Steve Glassey, Nicola Liebergreen, Marcelo Rodriguez Ferrere, Mike King

**Affiliations:** 1 Bioethics Centre, University of Otago, P.O. Box 56, Dunedin 9054, New Zealandmike.king@otago.ac.nz (M.K.); 2 Faculty of Law, University of Otago, P.O. Box 56, Dunedin 9054, New Zealand

**Keywords:** animal, disaster, evacuation, Edgecumbe, flood, pets, New Zealand, rescue, welfare

## Abstract

**Simple Summary:**

The 2017 Edgecumbe flood in New Zealand triggered the largest companion animal rescue operation in the nation’s history, highlighting significant gaps in disaster preparedness for animals. This study aimed to explore the experiences of six first responders from various agencies involved in the rescue efforts. The main challenges identified included the lack of logistical planning during the initial rescue phase, the need for proper animal identification and tracking systems, and the difficulties in decontaminating and providing long-term care for rescued animals. Health and safety issues for responders were also significant, with many facing hazardous conditions and emotional stress. This study concluded that comprehensive collaborative emergency response planning is essential to address the needs of both humans and animals. The findings underscore the importance of including animals in disaster preparedness plans and ensuring that responders are adequately trained and supported. These insights are valuable for improving future emergency responses, promoting community resilience, and ensuring the welfare of animals and humans during disasters.

**Abstract:**

The 2017 Edgecumbe flood in New Zealand necessitated the rescue of over 1000 animals, making it the largest companion animal rescue operation in the nation’s history at the time. This qualitative study explores the experiences and perspectives of six first responders from various agencies involved in the animal rescue efforts. Through semi-structured interviews, this study identified several key themes, including challenges during the rescue phase, post-rescue issues, the impact on the human–animal relationship and wellbeing, and the health and safety of responders. The rescue phase was characterised by difficulties in evacuating and rescuing animals, the influence of the socio-zoological scale on rescue prioritisation, issues with feeding animals in place, and the diversity of species requiring rescue. Post-rescue challenges included animal identification and tracking, decontamination, management of deceased animals, and long-term impacts on animals and owners. This study also highlighted the interconnectedness of human and animal welfare during the disaster, as well as the health and safety risks faced by responders. The findings underscore the need for comprehensive collaborative emergency response planning that addresses the needs of both humans and animals, as well as the importance of ongoing efforts to build resilience and preparedness in communities. Lessons learned from the Edgecumbe flood can inform future policy, planning, and practice to enhance the effectiveness and compassion of animal-inclusive emergency management.

## 1. Introduction

**Figure 1:**
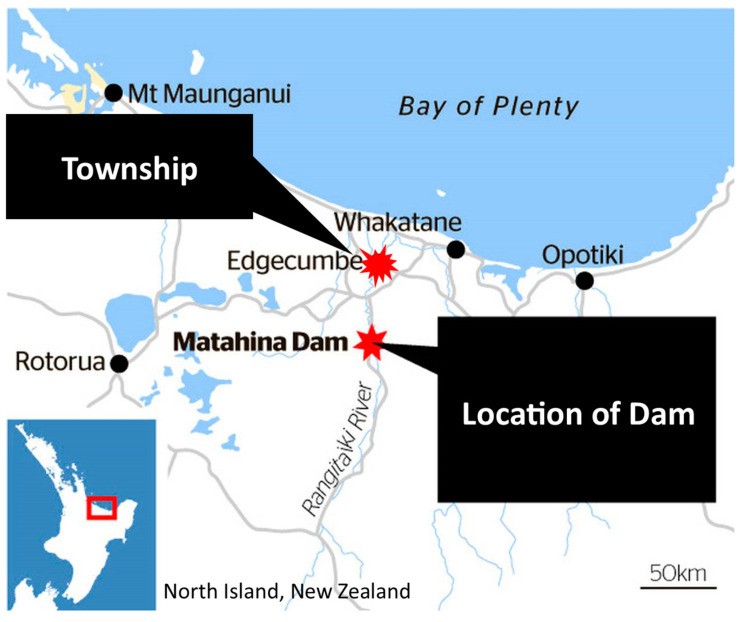
Map of the area.

On 6 April 2017, Cyclone Debbie’s heavy rainfall led to the Rangitāiki River overflowing its floodwall near College Road in Edgecumbe (Figure 1), a small New Zealand town. The flood struck at around 8:30 a.m., affecting a community of roughly 1638 residents across 597 households [1].

Prior to the incident, both the Bay of Plenty Regional Council’s flood management team and the Whakatane District Council’s civil defence organisation were actively monitoring the situation. The former had set up their flood room on 5 April, while the latter had activated its Emergency Operations Centre to coordinate the community response [2]. The Whakatane District Council, the municipal authority for Edgecumbe, declared a state of emergency at 12:40 p.m., several hours after the floodwall failure [2].

The breach’s proximity to residential areas resulted in rapid flooding, with water levels reaching up to 2 m in some locations. Residents had minimal time to evacuate, often leaving behind pets and possessions. In the aftermath, over 1000 animals were rescued, marking New Zealand’s largest companion animal rescue operation at that time [3]. A more detailed timeline is provided in Table 1.

This study provides perspectives from official responders involved in this large-scale animal rescue operation during the evacuation and the days that followed. It contributes to the global body of knowledge by providing a case study on mass animal rescue carried out by qualified responders in the context of a flood emergency. The outcomes are intended to create discussion and reinforce the need for improved animal welfare and emergency management arrangements, policies, and laws.

This event highlighted significant gaps in disaster preparedness, particularly regarding the management of various animal species—including pets, livestock, and wildlife—and their human caretakers during emergencies.

### 1.1. Emergency Management Arrangements

**Figure 2:**
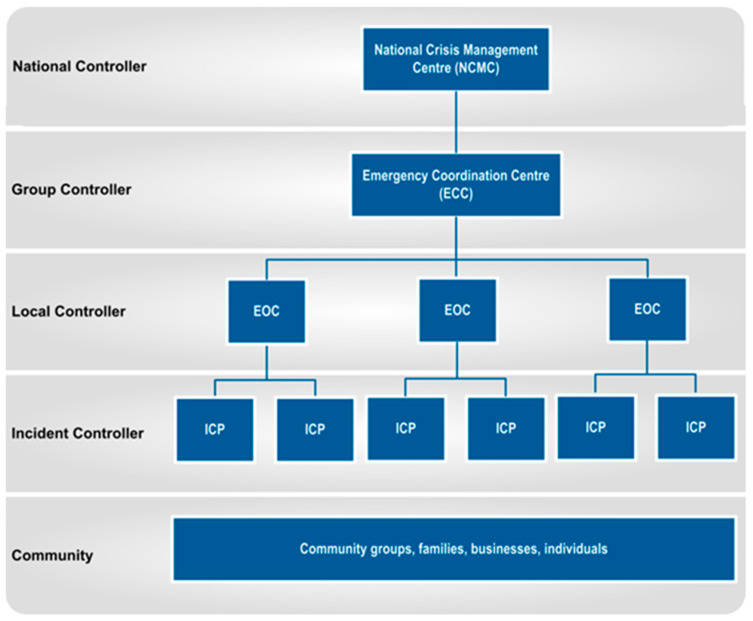
Emergency management structure during response phase [5].

Emergency management in New Zealand is structured under the Civil Defence Emergency Management (CDEM) framework, which was established by the Civil Defence Emergency Management Act 2002 (Figure 2). This framework emphasises a decentralised approach, where local governments play a critical role in fostering community resilience to disasters. Each region is required to form a CDEM Group, which consists of the regional council and the local territorial authorities within that region. These CDEM Groups are responsible for the comprehensive management of emergencies, encompassing risk reduction, readiness, response, and recovery, often referred to as the “four R’s” of emergency management. They are tasked with developing regional emergency management plans that align with the National Civil Defence Emergency Management Plan, with the intent of ensuring that local hazards, roles, and responsibilities are clearly defined and coordinated.

The National Civil Defence Emergency Management Plan includes mandating the Ministry for Primary Industries (MPI) to coordinate animal welfare including rescue at the national and group levels during civil defence emergencies, whereas local government animal management is mandated for the direct coordination of care, transport, and accommodation of companion animals where owners are unable to provide such responsibility. The National Civil Defence Emergency Management Plan, however, does not mandate any entity specifically for the delivery of animal rescue services or funding for such in a civil defence emergency.

Though the National Civil Defence and Emergency Management Plan in effect at that time had an evacuation principle that encouraged companion animals to be evacuated alongside their human guardians, this was not well applied, nor was it enforceable [4]. The Civil Defence Emergency Management Act 2002 (s.59) requires government departments and local authorities to undertake emergency management planning for their mandated functions. No emergency plan for animal welfare was held or in place at the time by the local authority, civil defence emergency management group, or Ministry for Primary Industries [4].

Local authorities, such as the Whakatane District Council and regional councils, are pivotal in the CDEM structure, providing the necessary resources and coordination at the community level. MPI plays an important role in managing emergencies that impact the agricultural sector, including animal welfare during disasters at the national and group level.

Fire and Emergency New Zealand (FENZ), formerly the New Zealand Fire Service, which it was known at the time of the Edgecumbe Flood, is another crucial partner responsible for fire-related emergencies and contributing to broader emergency responses. Entities like MPI and FENZ during a state of emergency are coordinated along with wider efforts by the Local Controller, who has significant statutory powers and does so from a designated facility known as the Emergency Operations Centre (EOC). As in the case of the Edgecumbe Flood, an incident control point (ICP) that provides on-scene direction was eventually established within the evacuated township to coordinate the animal rescue operation. Incident Control for the Edgecumbe Flood changed over time across the various phases of evacuation immediately following floodwall failure and the rescue of animals following evacuation [3].

### 1.2. Similar Studies

This study was conducted in parallel to two other studies on the Edgecumbe Flood led by Glassey, including an online survey conducted of event-affected households that considered the experiences of the community during and after the event [6] and a comparative analysis of a subsequent emergency in New Zealand impacting animals applying lessons identified from the Edgecumbe flood [4]. The household-level study found that most households (62.8%) received no instructions from officials on what to do with their animals. Where they did receive instructions, they were more likely to be instructed to leave them behind [6]. The study by Glassey et al. [4] compared action reports between the 2017 Edgecumbe flood and the 2019 Nelson wildfires, and found that only 7% of relevant lessons identified from the 2017 event were applied (learned) in the subsequent event that also impacted animals.

## 2. Methodology

Semi-structured interviews provided a meso-level analysis of the response, with participants being response agency representatives deployed in Edgecumbe as part of the flood event.

Purposive sampling methodology was employed to recruit people directly from the emergency response organisations involved in the Edgecumbe flood. Invitation letters or emails were sent to the key contacts from nine organisations/units asking them to nominate a suitable participant to be interviewed. This included: Fire and Emergency New Zealand (FENZ), New Zealand Police, New Zealand Defence Force, SPCA National Rescue Unit, SPCA Whakatane, Whakatane Emergency Response Team (NZRT17), Tauranga Response Team (NZRT15), Massey Veterinary Emergency Response Team (VERT), and Whakatane District Council Animal Management. Five organisations/units responded from which six people who acted as first responders during the 2017 Edgecumbe floods were interviewed.

### 2.1. Inclusion Criteria

To be included in the sample, the respondents had to be nominated by relevant public safety responder agencies and/or be known to have played a key role for that agency at the incident ground level during the Edgecumbe flood response. They also had to be over 18 years of age.

### 2.2. Interview Methodology

Interviews followed a semi-structured format, based around the following three lines of inquiry: what went well during the response; what could have been improved during the response; and what other reflections or observations could be shared to improve future responses.

Six first responders were interviewed from a cross section of response agencies, including the Society for the Prevention of Cruelty to Animals (SPCA), Fire & Emergency New Zealand (formerly New Zealand Fire Service at time of the incident), and New Zealand Response Teams (NZRTs).

The interviews were approximately 30–60 min long and were conducted via the Zoom online platform to ensure consistency of interview conditions and allow for recording. Recordings were transcribed and the transcripts were sent to the participants for their approval before analysis.

### 2.3. Ethical Considerations

There was a concern that the prior role of the principal researcher Steve Glassey, who led the rescue operation being studied, gave him an interest in this issue that could conflict with his interest in, and duty to, conducting an objective analysis of the data. This has been addressed in three ways; firstly, Glassey et al. incorporated a high degree of reflexivity into the methodology and followed conventional content analysis methodology. It is acknowledged that research cannot be value-free and this creates bias [7]. The reflexive position of this researcher allows for a greater in-depth understanding of the Edgecumbe Floods, but this is moderated through transparent data recordings (Zoom) and authorised manuscripts that prevent manipulation of results. The Zoom recordings were destroyed once the transcripts had been approved for use by the subject. Secondly, the qualitative data presented in this paper were double-coded by the second author in order to establish methodological rigor. Finally, the co-authors of this paper do not have any conflicts of interest as they played no role in, or had any connection to, the 2017 response, which acted to enforce objectivity.

Another area of concern was that recounting the events of the 2017 Edgecumbe Flood may have been distressing for some participants. It was made very clear that participation in this research was entirely voluntary and participants could withdraw up to the point of data analysis. All participants were provided with contact details for local psychosocial support services.

It was also recognised that the direct identification of participants in such a small community may pose a risk. To address this concern, during the consent process, the participants were asked whether they wished to remain anonymous. Two out of the six participants opted to be anonymous. Given the delay in analysis and to minimise the risk of identification of participants who sought anonymity, this study opted to anonymise all data to enhance ethical integrity.

## 3. Analysis

A conventional qualitative content analysis approach was used to analyse the data. In this article, we refer to Hsieh and Shannon’s definition of qualitative content analysis as “a research method for the subjective interpretation of the content of text data through the systematic classification process of coding and identifying themes or patterns” [8] (p. 1278). The purpose of this approach is “to provide knowledge and understanding of the phenomenon under study” [9] (p. 3140) and to allow the categories and their titles to emerge from the data [8]. All transcripts were read repeatedly, allowing each researcher to become immersed in the data and to develop a sense of the work as a whole [10]. Each transcript was then read word-by-word and the words that appeared to capture key ideas were highlighted and became the basis for the initial codes [10,11,12]. Notes regarding first impressions and initial analysis were made and coding labels began to emerge and were sorted into categories comprised of related codes. These emergent categories were then amalgamated into clusters [13,14] consisting of subthemes, and codes and definitions for each were developed. The first author used an Excel spreadsheet to track the coding development and provide exemplars for each code and category, and the second author used NVivo (version 14) software to code the data. These were then compared and reconciled.

The key advantage of this approach to qualitative analysis is that it allows the participants’ views to be explored, without imposing preconceived categories or theoretical perspectives on the data [8]. However, this method can result in the researcher failing to develop a complete understanding of the context and therefore overlooking key factors or categories [8]. Lincoln and Guba [15] define this as a credibility issue, and this has been addressed in this study through the use of peer debriefing, triangulation, and independent double coding of the data [15,16].

## 4. Results

**Figure 3:**
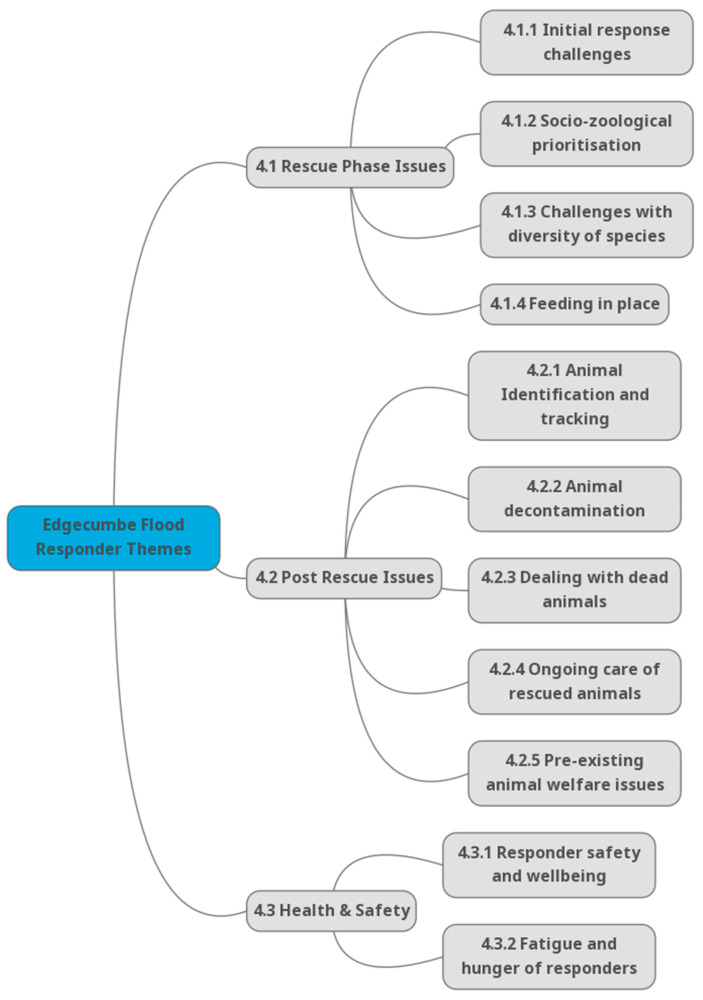
Responder survey themes.

The results revealed several key challenges faced by the responders. Three main themes emerged from the analysis (Figure 3): the difficulties encountered during the initial rescue phase (Section 4.1), the complex issues that arose after animals were rescued (Section 4.2), and the significant health, safety, and welfare impacts on the human responders (Section 4.3). These themes highlight the multifaceted nature of animal rescue operations during disasters and underscore the need for comprehensive planning and preparation to address both immediate and long-term challenges for animals, owners, and emergency personnel.

### 4.1. Theme 1: The Rescue Phase Was Challenging

#### 4.1.1. Challenges during the Evacuation and Rescue of Animals during the Initial Response

All participants reported that they faced many challenges in rescuing animals during and after the flood. When the teams first arrived on the scene, there was little logistical planning in place, and teams began the process by simply going from house to house;

*The first day we were there we would start at one house and work our way from house to house systematically checking each for animals. We ended up rescuing a couple of cats that had been locked in houses.*

(Responder 1)

During the initial evacuation stage, rescuers were dealing with both people and animals, each of which posed challenges;

*People can be stubborn and animals can be stubborn. You’ve got that on both sides. Then you have these others that want to get out, and some of the animals are like that too.*

(Responder 4)

In the first hours of the flood, some animal rescues were carried out by general emergency service personnel, rather than technical animal rescue specialists.

Responder 4, a Swift Water Technician, said they had trouble rescuing dogs as they were frightened and aggressive. As they were not skilled in animal handling, they were unable to handle them;

*We found a few dogs in the back of properties. Some would come and some others were quite aggressive. I would say very frightened. Obviously, they didn’t know what was going on and didn’t want to come to us. We just had to leave them there.*

(Responder 4)

During this first phase of the rescue operation, the speed at which people were evacuated left many animals in compromised situations. This meant that rescuing animals was often difficult and complex, particularly in the case of farm animals. Large animals, such as cows, presented significant logistical issues;

*We also rescued a couple of cows who were in the flood water. There was no dry land for them to get to. It was actually quite a mission to get a mother cow and her older calf that was with her out of this flooded area. There were fences everywhere and [we] couldn’t exactly see where those fences were located.*

(Responder 1)

*Me and a couple of the vets from Massey rescued some cows—different cows. There were originally six, one had died. We took some hay and went out the next day, one had died, and all the others looked hunched up. We were concerned that they would die if we left them there. From Google maps, I saw where the usual tracks are to the milking shed, so pushed them through chest deep water to get them out. I do believe if we hadn’t got them out, they would’ve died. That was pretty memorable, pretty excited about that.*

(Responder 3)

As time went on, systems began to emerge, and more tactical methods were utilised;

*I think we got into a pretty good flow from day three or four. Information came through from the EOC and MPI to our team. We broke up into smaller teams of two. We were assigned streets to check to see if we could find any animals. Through MPI, we had people who were evacuated ask us to check their house for animals that had been left behind. We would go to those locations to try and find their animals.*

(Responder 1)

#### 4.1.2. Socio-Zoological Prioritisation during the Response Phase

Responder 2 said that there were some discrepancies in the way rescuers viewed the needs of animals, which, at times, led to tensions around animal rescues and welfare;

*We had a couple of cross-over situations, for example Federated Farmers were in charge of the livestock as much as possible, however there were a few small herds of two to five cows within the town itself. There was an area there that had been contaminated with effluent waste and I wanted the stock to be moved, as an Inspector, because the grass they were eating and the water they were drinking was contaminated with faecal matter. Federated Farmers said to leave them there, that they would be fine! I was looking at it from an animal welfare point of view. I felt they shouldn’t be eating contaminated grass. They were in an easy position to move with a stock trailer. I kind of felt like someone could have dealt with them and they weren’t, because people had bigger fish to fry.*

(Responder 2)

While the welfare of dogs, cats, and most livestock was catered for by rescuers, other species were not seen as deserving of the same care;

*We had a lot of birds that needed to be rescued. Over 300 birds needed to be decontaminated, there was a huge range of very expensive exotics to the humble chicken, ducks and geese. One FENZ worker made [the] comment “It’s a duck, it can swim” and rolled their eyes at me. I tried to explain the animal is contaminated, however they thought my action was amusing.*

(Responder 2)

*We were getting goldfish. Massive large goldfish. People were like, “Why are you bringing in goldfish?” “These goldfish are worth 150 dollars each and this person cares about these goldfish like you care about your dog”.*

(Responder 2)

The lack of concern for some of the creatures by some agencies increased the workload for Responder 2, as people called her directly to come and help rescue their animals. Although a register system for missing animals was eventually put in place, some pet owners found this process frustrating;

*Some fish owners were frantically trying to contact us as they knew their fish would die without power, some went to the length of finding my own personal phone number to ring as they felt they were getting know where with the register.*

(Responder 2)

#### 4.1.3. Response Challenges with the Diversity of Animal Species

Responder 2 commented that the town of Edgecumbe differs greatly from an urban setting. People live closely with a variety of animals, and this provided challenges for the rescue teams;

*The thing with Edgecumbe that is probably unique about it is that there’s not just one animal on a property, there might be 12. They’ve got two dogs, five cats, seventeen chickens, four birds, and three rabbits a cow on their property. It’s a rural town and it’s what people are into.*

(Responder 2)

Responder 2 said they were working under great pressure and it was difficult to cater for all the animals’ needs when both time and resources were in short supply;

*We had to decontaminate the goldfish, take care of the goldfish, and give them the proper food. It was quite difficult getting every single species of animal into its own care system and we needed to find out what that was.*

(Responder 2)

Some farmed animals, such as rabbits, were also difficult to rescue due to the numbers involved and a lack of equipment;

*We went down to a rabbit farm and had to get all of their rabbits out. That was two days later after the flood. It was about forty rabbits, so we had to put them in whatever we could find. Load them up on the trailer and take them out. Then they took them somewhere else to [be] looked after.*

(Responder 4)

Responder 4’s team came across animals that needed rescuing while searching for people, and they had to do their best to rescue them with limited knowledge and resources;

*There were ducks and chickens, we saw a rabbit floating down on its hutch, so we got that out. Most of the animals, we just got the guys in the jet boats to see if they would go do something with them. We took the ducks down to the police and they took them to the fire station to start with, because we had nowhere for them to go until we could figure out what to do with them.*

(Responder 4)

Responder 4 alluded to the fact that the needs of the animals were perhaps compromised during the rescue, as it was difficult to house all the animals adequately;

*We stuck a couple [of rabbits] together and the owners were like, “Oh, they don’t like each other…” but oh well.*

(Responder 4)

On a positive note, some members of the community played a major role in housing recued animals;

*We had a lady who took the chickens on. She is really experienced and separated them into different areas so there was no cross contamination of animals coming from particular places. She has a talent for recognising them as individuals.*

(Responder 2)

As in most scenarios, the needs of people were prioritised over those of animals. People were rescued, but were not always allowed to bring their animals with them on the rescue craft. During the initial stages of the rescue operation, there was no plan in place for rescuing animals, so the emergency services did what they could and rescued any animals they found;

*We were looking for people at that stage. And once they said, “everyone is out,” along the way we started rescuing animals as well, because we started finding animals that had been left behind. I can’t even remember how long we did that for. It was quite a long day. Kind of followed the same pattern the next day once the river had settled down a bit.*

(Responder 4)

#### 4.1.4. Feeding Animals in Place

As roads were blocked and many of the town’s inhabitants were prevented from moving freely around the area, the welfare of some animals was compromised. This was particularly evident in the case of farmers wanting access to feed their stock;

*The farmers had huge issues at the roadblocks. They had some animals that were on the dry, that hadn’t been rescued yet. They were going hungry. They wanted to feed them. That’s all they wanted to do. They had tractors. All they wanted to do was feed their animals and get the hell out of there. The cops wouldn’t let them through. At one point, one particular roadblock there, there was going to be a big fisty-cuffs, so they had to send reinforcements.*

(Responder 5)

Thankfully, this situation was resolved quickly, and the animals were moved to a more accessible area;

*Common sense prevailed, they gave [the farmer] an escort, and the farmer was allowed to go in and do what he could do. [Over] the coming days they organised for these animals to come out via a safe route and be moved, and no longer be a part of the equation. That was really good.*

(Responder 5)

In addition to rescuing animals in Edgecumbe itself, people were also aware that some animals would be trapped with their owners in the wider area and have no access to food. Responder 2 worked with others to have bulk lots of feed delivered to these animals;

*[In] other places like Mount Manganui, myself and the Rotorua Inspector took three pallets of food up and we went door to door. The road was blocked and they couldn’t get into town for three weeks and they were feeding their animals possums, rabbits or whatever they could catch as they were completely isolated. We did two big food drops out there. They were really grateful.*

(Responder 2)

*In Ruatoki they couldn’t get in and out, so I did a food drop near there to their local Tuhoe Hauora and people would go to this facility to get the food when required. Animal Control also kindly took a load for us. They helicoptered it. There’s another little town along from Manganui where the road was totally blocked, so Graham from Animal Control put animal food on board with the human food drop via helicopter.*

(Responder 2)

### 4.2. Theme 2: Post-Rescue Phase Issues

#### 4.2.1. Animal Identification and Tracking

In order to keep track of all the rescued animals, systems had to be developed quickly and carried out efficiently. Responder 2 said that this was done well and animals were identified and tracked in an effective way;

*We had really good responsible people on the ground and the (NZ)RT-17 ladies doing logistics, so there were no muck-ups really. Of all of those animals that came through, we knew who they were, where they came from, and where they were going to go to, so that’s what went really well.*

(Responder 2)

Responder 2 said that, while micro-chipping animals is the ideal identification method, this was not practical for several reasons and other methods had to be developed;

*[The field] wasn’t a secure place to microchip them. They were quite stressed in those cages and a lot of the animals that wouldn’t [normally] bite, would bite if you tried to put your hand in to chip them. And when they got to the centre, I didn’t have staff that could do it. At the centre, I am the only one that is verified to microchip. I couldn’t be there to do that.*

(Responder 2)

Responder 2 was also worried that microchipping without consent was a manipulation of animals and she wanted clarity around that before implementing this procedure;

*The worry here for me also was us microchipping without consent is a manipulation of that animal and I wanted clarity around this before we did it.*

(Responder 2)

Due to these issues, Responder 2 instigated a manual labelling system, which she said worked well under the circumstances;

*The labels on the cages were the best thing. So we just shifted the label from the cage to the transporter cage or to the secure cage. It just followed wherever the animal went. And we checked the register. “5 ducks from 22 Rimu Street went to Bird Rescue Veterinary Centre” and so on.*

(Responder 2)

#### 4.2.2. Decontamination

As many of the rescued animals were found swimming in filthy water, decontaminating them was important from a welfare point of view. This was difficult given the volume of animals rescued and the small team of people available to perform the work. This led to a triage system being developed that prioritised the most urgent cases;

*The VERT (Veterinary Emergency Response Team) team were amazing regarding the birds and had a bird specialist on board. The recommendation was to decom every bird, for good reason, however to do this was feeling impossible, so once the birds got to the clinic the bird team and vets asked me if they had to decom everything. I said to triage and prioritise and do what they could.*

(Responder 2)

#### 4.2.3. Dealing with Dead Animals

Sadly, not all animals were able to be rescued and there were a significant number of fatalities. Responder 2 said this was difficult, as many of the people working during the flood were volunteers and they were not prepared for dealing with dead animals.

*Volunteers don’t want to see that. In their mind the SPCA or rescue agencies are there to rescue animals. It’s quite traumatic for them. I mean it’s probably traumatic for me, but I’m used to dealing with seeing yucky stuff all the time. I know it’s cumulative. I know how to go away and download and get rid of it, where they don’t. It’s not something they see all of the time. Even though we try to make it private and away from everybody else, it’s difficult, they know what’s happening. Especially, when we have a chest freezer full of dead animals.*

(Responder 2)

As someone who had experience in dealing with dead animals, Responder 2 spent considerable time and effort to make the animals more presentable for their owners so as to reduce the distress as much as possible;

*One area that new volunteers find difficult to deal with is the dead animals coming in. For example, we’d find a dead body, bring it back to the centre, and I would have to clean it and make it look more nice and tidy so that the owner could view it. That’s really difficult. Not many could do it. I ended up doing a lot of it. I came in from the field at a certain time to do this. Some of the animals were so bad that I had to sit there and wash them for half an hour in hot water to tidy them up. Defrost their face and wrap them up in a towel, blow dry their face so they are presentable. You can’t have people seeing their pet how we found it.*

(Responder 2)

Responders 1 and 3 both commented that there were many deceased birds to deal with and Responder 1 also said that many fish had become trapped and died;

*The force of the water when the stop bank was open was such that animals, unless tied down, were washed away. Most animals I did see deceased were the birds. Edgecumbe has an unusually large population of aviary birds and tropical birds, which just didn’t fare well. Quite a few chickens didn’t fare well too. Chickens float initially but are not designed to be in water for extended periods. Saw quite a few of those.*

(Responder 3)

*We came across a lot of birds that did not survive the event. There were free range chickens in the yards, bird in the aviaries. We also saw rats, some dead rats. Fortunately for me, I didn’t see any dead cats or dogs. There were a lot of fish as well. I do not know how they survived in muddy ponds. We even came across fish trapped in gutters. Fish that got out of their pond and ended up in a pond somewhere else.*

(Responder 1)

In order to keep deceased animals cool while their owners were located, a temporary morgue was established by the SPCA;

*…we had [a makeshift morgue]. Most food chillers are used for food and you’re not allowed to use them for anything else, we had to order a special chiller from Tauranga to put animal bodies in. Once dead, animals are classed as toxic waste and need to be handled as such, however being mindful they are a family member, we had to find a happy medium to deal with this. We put the chiller behind an industrial building away from everyone else, so they couldn’t see it and know it was there.*

(Responder 2)

Responder 2 was very aware of the distress the loss of a loved animal could cause people, so she arranged for discreet access to the animals;

*Depending on the situation, I’d bring the animal to the centre. Clean it up and tidy it up and then take it over there where the morgue was. So, the owners and animals were not in view to people coming in and getting their live animals from the centre.*

(Responder 2)

She also arranged for emotional support for people visiting or collecting their deceased animals;

*We got the Salvation Army Chaplain and an animal grief counsellor to be available for the owners to support them when they came to retrieve their passed-away family members.*

Responder 2 said she witnessed a range of different reactions from people who came to farewell their animals and she had to respond appropriately;

*We had a mixed range of responses from deceased pet owners from, “We can’t see it” to “Can we have a cremation?”, to a large Māori family stepping out of their vehicles and nanny started calling a very loud, moving tangihanga from across the road as they walked up the path to retrieve the body of their dog. The whole family followed in procession and performed a waiata (indigenous prayer) before they took the dog home.*

(Responder 2)

Responder 2 went to considerable lengths to make the loss as easy as possible for the families she dealt with. Responder 2 said she felt this was part of her duty as a member of the SPCA;

*A few animals were a bit too messed up, but the ones that we could, we tried to make the effort. It’s a member of their family. There are some people that think it’s over the top and you don’t need to do that. I felt that we needed to do that and deal with people’s psychological and emotional needs. Especially, when we’re an organisation like the SPCA, if we put our hand up to do the job we might as well do a good job of it.*

(Responder 2)

#### 4.2.4. Many Animals Required Ongoing Care after the Rescue

Once the initial rescue phase was over, things began to settle in Edgecumbe; however, there were many displaced people and accordingly, many displaced animals. These animals required long-term care, sometimes for many months, and this posed a number of challenges.

Responder 2 said that many animals required care from fosterers, as their owners were not able to care for them after the flood due to their living situations or other factors. While she was grateful to many of the volunteer fosterers, the SPCA did not know all these people or have control over their behaviour. The SPCA were aware of the danger of negative outcomes or complicated situations, so they took steps to distance themselves from the fostering arrangements;

*…we tried to stay out of the foster situations. Once they got to us, we were like, “Hey, we have fosterers that have rung and volunteered to take animals, but you have to be responsible to ring them and make the arrangements for your pets, the arrangement is between you and them and does not include us. […] We are happy to support with food, but anything else has nothing to do with us”.*

(Responder 2)

Responder 2 was also aware that people sometimes volunteer to look after animals without thinking through the long-term commitment they are making;

*[People] just put their hand up [to foster animals] at the time. The owner had arranged it himself. I think a lot of people do that and don’t understand the actual long term and the consequences of having that animal for that period of time. The owners are stuck between a rock and a hard place. They have no home and it might be two months away or two years away. They don’t know.*

(Responder 2)

As time went on, difficulties with fostering began to arise and were brought to the attention of the SPCA, who sometimes had to intervene;

*…people would foster for other people in good faith, but the animals started to suffer because they’re on a chain 24/7. Fosterer can’t afford to feed them and they are over it, they’re not walking them anymore, so we started having welfare concerns for those animals. I would recognise those animals from Edgecumbe when I would get there. A property I went to, a bunch of guys were all standing there and I said, “That was the dog in the paper with me”, and they were like “Yeah yeah, we’re looking after it for a mate from the floods.” He was looking after it for a friend but had no money and the dog was in poor condition. It had been on a chain for a month or two.*

(Responder 2)

*We had a bitch come in and I recognised her because she had a cruciate type injury. At the time of the flood we were so busy that we couldn’t get her looked at. She wasn’t critical. She went back to the owner and I was called to the welfare complaint and same thing; she was fostered out because the owner had no house. She was living in a paddock by herself. Still had this leg injury. The family offered to foster it but they gave it a hiding because it was bothering the cat and killing the chickens. They tied her up in the middle of the paddock next door by herself with inadequate shelter, food or water. I went and got her and took possession of her under the Animal Welfare Act. They eventually signed her over and we had her leg amputated and rehomed her.*

(Responder 2)

Another long-term issue was that a number of dogs were roaming free, as their owners had lost fencing in the floods. Responder 2 said that Animal Control took a pragmatic view of this, and that residents needed to be patient while the infrastructure was rebuilt;

*If you look at the Kia Kaha Edgecumbe Facebook page, there’s people complaining that someone’s dogs are roaming. I know that [name] from Animal Control’s point of view would be understanding around this issue, due to the flood damage, particularly if the animal is not causing an issue. So even though some residents may not be happy about it, they’ve got to understand the big picture.*

(Responder 2)

Responder 2 and her colleagues also had to deal with the emotional issues both owners and fosterers faced in coping with animals that were moved between carers;

*I had issues with foster carers not wanting to release the animals back to their owners after three or four months.*

(Responder 2)

Conversely, some owners were not in a position to reclaim their animals for many months, leaving fosterers with a much longer commitment than they had expected;

*The owners weren’t going to collect their animals, and some fosterers eight months down the track were still looking after flood animals.*

(Responder 2)

The SPCA also had to step in and suggest euthanasia for some animals as their welfare was being compromised. This was difficult to negotiate as the owners were often in stressful situations themselves and euthanising their animal was a very difficult decision to make;

*We also had to assist five owners, talking to them about euthanising their animals, because they were not coping with the stress or were aged or had medical problems and they couldn’t cope with the situation they were in. The owners were still 12 months away from actually having a home or weren’t sure when they had a residence. This was quite heart wrenching. We had to talk with them about what options were available in case they didn’t have money for behaviourists and those sorts of other things. What was best for the animal overall? We would pay for them and be with them when that process happened if they wanted, or we would do it for them delivering the animal home where required. My manager and I were the ones that dealt with these delicate issues.*

(Responder 2)

Those animals that were cared for by boarding facilities also faced health issues as a result of being kept with other animals. The boarding facilities themselves had to deal with unexpected outbreaks of disease due to boarding unvaccinated animals. This was financially challenging and discouraged these facilities from offering to take animals in emergencies in the future;

*Boarding kennels had an issue with giardia from the flood animals and it spread right through their businesses. Understandably they weren’t happy. The contamination took them a while to sort out. There was no financial assistance for them, and they had to financially sort it out themselves. This obviously includes their loss of income as they had to quarantine their facilities. I’ve had some feedback from a couple of the vets and one of the boarding kennels saying they’d never do it again. The disease issue became quite prevalent following the floods with an increase in Leptospirosis, parvo, fungal and bacteria skin issues.*

(Responder 2)

Another long-term issue was that, once people returned to their homes many months later, they would find animals living and breeding there, requiring the SPCA to collect the animals and try to rehome them;

*…it was about five months later and people were going back to their houses and finding cats with kittens. We would take pictures of them and post them on Facebook and ask, “Is this your cat?” however very few people claimed them, and we then had the issue of trying to find homes for these animals.*

(Responder 2)

Another issue facing the SPCA and others helping to rescue animals was that the workload resulting from the flood was huge and continued to stress their operations for months afterwards. Responder 2 said that this reduced her capacity to work as an inspector because she was required to assist with the work usually carried out by her manager;

*[It took] four and half months at least [to return to a normal level of operation]. Even after I left, a year later, they were still dealing with some of the stuff from the floods…*

(Responder 2)

Similarly, not only did poor fostering outcomes have terrible consequences for the animals affected, but also for animals more broadly, as the SPCA shouldered a great financial burden as a result;

*There were other welfare issues like they’d taken a foster dog on that has fought. Same thing, the family has done the kind-hearted thing, but don’t have the money to deal with the medical costs of dogs attacking or fighting so we’ve come in and covered that as well, just to keep the peace and to keep everybody happy. All in all, SPCA took on quite a financial burden.*

(Responder 2)

*We did pay out of the SPCA pocket a number of desexings and for the animal to stay somewhere else because the animals were fighting or not coping well.*

(Responder 2)

#### 4.2.5. Pre-Existing Animal Welfare Issues Complicated the Rescue Effort

While most of the animals that were rescued were well looked after, some animals had existing welfare issues that made them even more vulnerable once the floodwaters hit. This was compounded by the fact that some owners did not want to alert rescuers to their animals’ plight, as they were scared of repercussions;

*I investigated the situation and contacted rescue unit 17 and said, “You guys have got to get me in there”. We got all of the animals out in poor condition. I hadn’t left an inspector’s note due to circumstances, however I was in contact with the owner and formally interviewed him later in the process. One dog was in labour and we had to give it an emergency caesar. She survived but the pups didn’t.*

(Responder 2)

*Then there were four pig dogs in their kennels up to their necks in water. I think they had been there the whole time of the flood—three days. But they hadn’t been fed five to six days prior to that. I was amazed they were still alive. They were in very poor condition. The bitch was whelping, so we got them all out via the house including two love birds to the back of the ute and drove out and went straight to the clinic. I dealt with that guy afterwards. He admitted to not knowing how to look after the dogs, as they were his brothers who was in jail. We paid for the bitch to have her surgery done.*

(Responder 2)

### 4.3. Theme 3: Health, Safety and Welfare Impacts on Humans

#### 4.3.1. Health and Safety Issues for Rescuers

Health and safety issues were mentioned by several participants and many alluded to the difficulties of working in a flooded environment;

*[The water was] very swift the first day. It was like being on the river. We couldn’t swim in it. We got around the corner and in some of the cul-de-sacs it was okay, but where it was coming into Puriri Crescent, it was flowing out of the back of Matipo Place, which was literally like a river. And the only way to go around was to go through the back of people’s properties or jumping on the back of the jet boats and they would take us across the street. Otherwise we would’ve been swept down into the paddock, it was pretty swift.*

(Responder 4)

Due to the contaminated water, there was concern about people being exposed to dangerous substances;

*On the first day, the water was mainly mud and soot and debris. That sort of stuff and insects. One street in particular had gasoline and oil and other things of that nature in it. This street remained flooded for a long time compared to some of the other areas the day after we had arrived. You could see the oil on the top and smell the gasoline. I’m also relatively confident that there was sewage in the water at this location. Just the colour of it. It wasn’t a nice muddy colour.*

(Responder 1)

*We came back more or less to make sure the guys were using the right PPE and were alright, doing our own paper trail and deployment documentation. I was very concerned about the guys being in potentially contaminated areas not using the correct PPE.*

(Responder 6)

Those rescuing animals faced additional risks, as they were dealing with frightened animals that sometimes lashed out at rescuers;

*That could potentially put the responder out of action because he gets bitten or he got scratched by a cat. “Oh, it scratched my face and I can’t go there because of contaminated water.” So taking the steps prior and trying to minimise those accidents [was important].*

(Responder 5)

Responder 1’s team had lost their vehicles containing their personal equipment, so they were left without clean clothes and toiletries. This “added in a different level of stress”, which was partly alleviated with the Incident Controllers’ help;

*… we went back to the Whakatane response team’s headquarters where we washed our overalls and had some dinner. We also were able to debrief on the day. Unfortunately, a lot of our personal gear and equipment was ruined when the vehicle flooded. Most of the team didn’t have clothes to change into, a toothbrush or anything. This added in a different level of stress, which we all managed to get through. Our Incident Controller organised for us go over to Kmart and buy some undies and basic things that we needed so we could recover overnight and get back into it full swing the next morning.*

(Responder 1)

Other rescuers were faced with scenarios where they had to risk their own physical safety to reach the trapped animals;

*There were five dogs. I had to climb out the rescue truck window onto a car roof to get to the deck of the house as it was up to its windows in effluent. Went through the house to the back of the property. They had drugs there and it was all penned off and locked up, so you couldn’t get in. It was up to probably the bottom windows in sewage and the inside floor had sewerage soaked through the carpet. The effluent was so deep it wrecked the rescue vehicle because all of the sewage and crap went up into the airbags.*

(Responder 2)

#### 4.3.2. Rescuer Fatigue and Hunger

Several participants commented on the long hours worked and the relentless nature of the tasks they were faced with. This was compounded by the fact that many of them had to travel considerable distances each day;

*As far as team welfare, working an entire day, then having a long drive was not ideal. We didn’t have enough people to properly share the drive. In the future, we would not do that again. People need to be rested in order to function well in response environments.*

(Responder 1)

Responder 2 said that it was also difficult organising team rosters as people were tired, but were sometimes unwilling to be stood down. Responder 2 said this was extremely difficult to negotiate;

*When people became fatigued, they started complaining, which was fair enough, however they wouldn’t step down either to allow others to come in. With assistance from [redacted] at National [Office] we prepared a fresh team to come in, experienced inspectors, auxiliary officers from other centres to take over and give everyone a break. But they wouldn’t let go. I felt a bit damned if I did and damned if I didn’t. I felt quite a bit of angst there as I wanted to protect and respect my team whom had done an awesome job, but they needed to stand down.*

(Responder 2)

*I think trying to keep that morale up and having a good change of people at the right time will alleviate this issue. I know even after being there for ten days straight doing 18 h days myself and when Cyclone Cook came through I had to spend it under my desk for safety. My truck was hit by a tree when I was trying to get to the centre for safety, billboards flying past at roof height. I started getting panicky and worried, but I knew it was because I was tired. Your response to things is different when you’re in that headspace and you’re mentally tired and your body’s tired. While mentioning bodily functions the food made and gifted for the volunteers was lovely however functioning on chocolate, cake, biscuits, sausage rolls is not sustainable and leads very quickly to tired weary bodies. I am aware there was not much handy at the time but being prepared for this would assist greatly from experience. I couldn’t get home, nothing was open so spent a few nights eating a biscuit here and there to keep going.*

(Responder 2)

Responder 3 said they faced many challenging situations, and she was pleased with the performance of her team;

*The evacuations weren’t simple. It wasn’t something where you could just pick up and go. Some of them were, but some of them really required a lot of thought and planning and thinking outside of the box. …The team showed real skill and perseverance in that regard.*

(Responder 3)

## 5. Discussion

The results of this study indicate that there are considerable lessons to be learned. Many of these have been identified in other studies, both in New Zealand and abroad, such as flood contamination, responder safety, stress impacts on persons involved or affected, animal recording systems, lack of capability and capacity, and lack of emergency planning [17,18,19]. The unexpected results from this study highlighted challenges with the unstructured fostering of flood-displaced companion animals, management of deceased companion animals, socio-zoological prioritisation for rescue, and reluctance of animal boarding facilities to be involved in future emergencies.

### 5.1. Socio-Zoological Scale

The concept of the socio-zoological scale, which ranks animals according to their perceived importance in human society [20], was evident in the rescue prioritisation during the Edgecumbe flood response. Responders noted that, while the welfare of dogs, cats, and most livestock was catered for, other species, like birds, fish, and “exotic” pets, were sometimes overlooked or dismissed by rescuers. This selective rescue effort reflects societal biases towards certain animal species and highlights the need for a more inclusive and equitable approach to animal rescue in disasters [21].

Responder 2’s account of a worker from human-centric emergency service dismissing the need to rescue ducks because “they can swim” and people questioning the rescue of expensive goldfish demonstrates how the socio-zoological scale can influence rescue decision-making. It is important for emergency responders to recognise the intrinsic value of all animal life and the strong bonds that can exist between humans and their companion animals, regardless of species [22]. Training and awareness programs for responders should emphasise the importance of treating all animals with respect and providing appropriate care based on their needs.

### 5.2. Feeding in Place

The concept of “feeding in place” emerged as a significant challenge during the Edgecumbe flood response. With roads blocked and access restricted, many animals were left without food and water for extended periods. This was particularly problematic for livestock, as farmers struggled to reach their animals to provide feed, as experienced in other flood events reported by Paulik et al. [23] and Smith et al. [24]. It was apparent that many animals died unnecessarily while waiting to be rescued due to decision makers declining the request for additional specialist rescue resources.

The confrontation between farmers and police at roadblocks highlights the tension between human safety concerns and animal welfare needs in disaster situations. While it is understandable that authorities want to restrict access to flooded areas for safety reasons, there must be provisions in place to ensure that animals can receive necessary care [24,25,26]. This could include establishing protocols for escorted access for farmers to feed and move livestock or setting up temporary feed stations in accessible locations.

The SPCA’s efforts to deliver bulk lots of feed to isolated areas and coordinate with other agencies to include animal food in helicopter drops demonstrate the importance of collaboration and resourcefulness in meeting animal welfare needs during disasters. Emergency response plans should include strategies for providing food and water to animals in place, as well as contingencies for evacuation if necessary [25,27].

### 5.3. Diversity of Animal Species Needing Rescue

The Edgecumbe flood highlighted the wide diversity of animal species that may require rescue in a disaster situation. In addition to the more commonly recognised companion animals, like dogs and cats, responders encountered birds, fish, rabbits, and livestock in need of assistance [28].

Responder 2 noted that Edgecumbe differed from an urban setting in that people often lived closely with a variety of animals, presenting unique challenges for rescue teams. Rescuing and caring for such a diverse array of species requires specialised knowledge, equipment, and facilities [29]. Responders may not always have the necessary expertise or resources to handle every type of animal they encounter.

This underscores the need for emergency response plans to include provisions for a wide range of animal species and to involve personnel with relevant expertise [30]. Collaboration with organisations like the SPCA, animal control agencies, and veterinarians is crucial to ensure that all animals receive appropriate care. Training programs for responders should cover basic handling and care techniques for common animal species likely to be encountered in disasters.

The rescue of fish, in particular, presented challenges due to their specific habitat and care requirements. Responder 2’s account of having to decontaminate and find proper housing for over 300 birds and fish illustrates the logistical difficulties involved in caring for these species in an emergency setting. Research by Parker-Graham et al. [28] documented comparable difficulties in rescuing and evacuating pet fish during various disaster scenarios, particularly noting the challenges faced during the California wildfires of 2017 and 2019. Emergency animal shelters must be equipped to provide suitable environments and care for a variety of species [25,31], and fish have specific water quality and handling requirements that are often not well understood [28].

### 5.4. Identification and Tracking of Rescued Animals

Proper identification and tracking of rescued animals emerged as a critical issue during the Edgecumbe flood response. With large numbers of animals being rescued and transported to various locations, it was essential to have systems in place to keep track of each animal’s identity, origin, and destination [25,32].

Responder 2’s account of the SPCA’s labelling system for animal cages highlights the importance of having a standardised method for identifying and tracking animals during a disaster response. By using labels that followed the animals wherever they went and cross-referencing with a central register, the SPCA was able to maintain accurate records of all rescued animals, despite the chaotic circumstances. However, this was agency-specific and not on a shared platform. The need for a standardised animal registration and tracking system was identified in the post-event report published nearly four years after the event [33] and in other reports from subsequent emergencies, such as the Nelson Fires [34].

However, Responder 2 also noted challenges with implementing microchipping as an identification method during the emergency. While microchipping is an ideal long-term solution for animal identification, it may not always be practical or safe to perform during a disaster response due to animal stress, lack of resources, and time constraints [25].

Companion animal microchipping in New Zealand has achieved widespread success, largely due to the implementation of a streamlined system utilising only two centralised databases. These databases are operated by Companion Animals New Zealand and the Department of Internal Affairs, with the latter exclusively dedicated to dog control purposes. This centralised approach stands in stark contrast to the situation in the United States, where no nationally adopted animal microchip database exists. The fragmented nature of animal welfare organisations in the U.S. presents a significant barrier to achieving a unified system, as consensus among these diverse entities would be required [17].

Emergency response plans should include multiple methods for animal identification and tracking, such as temporary identification tags, photographs, and written records, in addition to microchipping when feasible [25,32]. Responders should be trained in proper documentation procedures to ensure that all relevant information is captured and communicated effectively between agencies.

### 5.5. Decontamination of Rescued Animals

The Edgecumbe flood resulted in many animals being exposed to contaminated floodwater, necessitating decontamination procedures to protect their health and welfare. Responder 2’s account of the challenges involved in decontaminating over 300 birds and fish highlights the scale and complexity of this task.

The decontamination of animals exposed to floodwater is crucial to prevent the spread of disease and ensure their wellbeing. However, it can be a time-consuming and resource-intensive process, particularly when dealing with large numbers of animals. Emergency response plans must include protocols and resources for animal decontamination, including appropriate cleaning agents, personal protective equipment for responders, and designated decontamination areas [35,36]. Beyond natural hazard events, such as floods and earthquakes, the lack of animal decontamination has been highlighted in numerous after-action reports shared with the World Organisation for Animal Health (OIE). These reports emphasise the importance of such capacities and technologies during emergencies like Anthrax terror attacks, which continue to be a commonly identified weakness in reviews [37].

Triaging animals for decontamination based on their level of exposure and risk is necessary when resources are limited. Responder 2’s decision to prioritise the most urgent cases when faced with the overwhelming task of decontaminating every bird demonstrates the type of difficult judgement calls that responders must make in crisis situations.

Training for responders should cover proper decontamination techniques for different species and scenarios, as well as strategies for managing limited resources and making triage decisions [38]. Collaboration with veterinary professionals is essential to ensure that decontamination procedures are safe and effective for each species [38].

### 5.6. Dealing with Deceased Animals

Sadly, not all animals could be saved during the Edgecumbe flood, and responders had to deal with the grim task of managing deceased animals. This included recovering bodies, identifying remains, and providing respectful storage until owners could be notified.

Responder 2’s account of the emotional toll of cleaning and preparing deceased animals for viewing by their owners highlights the need for sensitivity and support for both responders and affected community members in these situations. Dealing with animal remains can be traumatic, particularly for owners who have lost beloved pets.

The establishment of a temporary animal morgue by the SPCA was a crucial step in managing deceased animals with dignity and respect. However, Responder 2’s description of the challenges involved, such as finding an appropriate location and maintaining proper temperature control, underscores the need for advance planning and resources for this aspect of disaster responses.

Just as there are established protocols for human disaster victim identification (DVI), there is a need for a similar process for deceased animals in disaster situations. This could include standardised methods for documenting and identifying animal remains, as well as guidelines for their respectful handling, storage, and disposal [39].

Training for responders should cover the psychological impacts of dealing with deceased animals and provide strategies for self-care and supporting affected community members. Collaboration with mental health professionals and grief counsellors can be valuable in providing support for both responders and owners.

### 5.7. Long-Term Impacts on Animals and Owners

The Edgecumbe flood had significant long-term impacts on both animals and their owners during the recovery phase. Many animals required ongoing care and support for months after the initial rescue, while their owners struggled to find suitable housing and care arrangements. The lack of pet-friendly rental accommodation following disasters has been a common occurrence, often forcing owners to relinquish, rehome, or euthanise their pets [40]. This can negatively impact human and animal wellbeing and resilience [41,42]. In some cases, owners may move to unsafe neighbourhoods to avoid pet relinquishment, which can increase their vulnerability [41]. For example, following the 2014 floods in Calgary, some pet owners relocated to higher-risk areas to keep their pets, potentially increasing their vulnerability [41].

Responder 2’s accounts of the challenges with long-term fostering, such as animals being returned in poor condition or owners being unable to reclaim them, highlight the need for better planning and support for animal care in the aftermath of disasters. Emergency response plans should include provisions for transitional and long-term animal housing [26], as well as resources for owners who may be displaced or facing financial hardship.

The emotional toll of the flood on both animals and owners was also significant. Responder 2 described cases of animals suffering from behavioural issues and stress due to the upheaval, as well as owners struggling with the decision to surrender or euthanise animals they could no longer care for. These impacts underscore the importance of considering the long-term mental health and welfare needs of both animals and humans in disaster recovery planning [21,42,43].

Support services for owners, such as counselling, financial assistance, and help with finding pet-friendly housing, can be crucial in the months following a disaster. Collaboration with human services agencies and community organisations is essential to ensure a holistic approach to recovery that addresses both human and animal needs.

### 5.8. Pre-Existing Animal Welfare Issues

The Edgecumbe flood also exposed pre-existing animal welfare issues and gaps in animal health infrastructure that compounded the challenges of the disaster response. Responder 2 described cases of animals that were already in poor health or living in substandard conditions before the flood, making them even more vulnerable when the disaster struck.

These pre-existing issues highlight the need for ongoing efforts to improve animal welfare and strengthen animal health infrastructure in communities, particularly those at high risk for disasters [44]. This could include initiatives to increase access to veterinary care, promote responsible pet ownership, and enforce animal welfare regulations.

Collaboration between animal welfare organisations, local government agencies, and community groups is essential to identify and address animal welfare concerns before disasters occur. By building a strong foundation of animal health and welfare in communities, we can improve the resilience of both animals and their owners in the face of future crises.

### 5.9. Evacuee Health and Mobility Challenges

In addition to the challenges facing animals, the Edgecumbe flood also highlighted the difficulties faced by human evacuees, particularly those with health conditions or mobility impairments. Responder 5 described cases of evacuees who had to leave behind essential medications, mobility aids, and medical equipment in the rush to evacuate, compromising their health and safety.

Similar studies have also found that pets, medications, and important documents were drivers for evacuees to return to cordoned homes following a disaster, such as in the 1997 Yuba County flood [45] and 2010–2011 Christchurch earthquakes [42].

This underscores the need for emergency response plans to include provisions for the specific needs of vulnerable populations, such as the elderly, disabled, and those with chronic health conditions. Evacuation protocols should ensure that these individuals have access to necessary medications, equipment, and support services throughout the evacuation and recovery processes.

Collaboration between emergency responders, health care providers, and community organisations is crucial to identify and assist vulnerable individuals in disaster situations. Training for responders should cover strategies for safely evacuating and supporting people with diverse health and mobility needs.

### 5.10. Responder Injury Trends and Safety

Responding to the Edgecumbe flood also posed significant risks to the health and safety of the responders themselves. Responders faced hazards such as contaminated floodwater, debris, and unpredictable animal behaviour, leading to potential injuries and exposures [3].

Responder 1’s account of losing personal protective equipment when their vehicle flooded highlights the importance of ensuring that responders have adequate supplies and backup resources to maintain their safety throughout the response. Emergency response plans must prioritise the health and safety of responders, including providing appropriate training, equipment, and support.

Monitoring injury trends among responders can help to identify areas where additional training or resources may be needed to prevent future incidents. Regular debriefing and health monitoring can also help to identify responders who may need medical attention or psychological support following the response.

Animal rescue workers face significant risks of both physical and mental injuries during disaster response efforts. Physical hazards include exposure to dangerous conditions, such as floods, fires, and debris, which can lead to injuries like lacerations, fractures, and infections [46,47]. Rescuers may also be injured by panicked, injured, trapped or aggressive animals [32,46,47,48].

The mental health impacts can be even more severe. A study by Vroegindewey and Kertis found that 51% of veterinary workers from across the Asia–Pacific region, Africa, Europe, Latin American, and South America who responded to disaster events reported behavioural health issues during and up to 6 months after the event [47].

Comprehensive emergency planning is critical to mitigate these risks. This includes proper training, protective equipment, mental health support systems, and clear euthanasia protocols [25,48]. Integrating animal welfare into broader disaster response frameworks can also improve coordination and outcomes for both human and animal rescuers [25,27,32,39,49]. As climate change increases the frequency and intensity of disasters, addressing the wellbeing of animal rescue workers will only become more urgent.

### 5.11. Responder Fatigue in Flood Operations

The Edgecumbe flood response highlighted the significant risk of fatigue and burnout among responders during prolonged disaster operations. Responders described working long hours in challenging conditions, often with limited rest and support.

Fatigue can impair responders’ physical and mental performance, increasing the risk of errors, accidents, and poor decision-making [50]. It can also have long-term impacts on responders’ health and well-being, including increased risk of chronic stress, anxiety, and post-traumatic stress [47,50].

Emergency response plans must include strategies for managing responder fatigue, such as establishing appropriate work–rest cycles, providing adequate nutrition and hydration, and ensuring access to mental health support [47,50]. Supervisors should monitor responders for signs of fatigue and take appropriate action to ensure their safety and well-being [47,50].

Responder 2’s account of the challenges of managing team rotations and encouraging fatigued team members to step back highlights the importance of having clear protocols and leadership support for fatigue management. Training for responders should cover strategies for self-care and recognising signs of fatigue in themselves and their teammates.

## 6. Limitations and Future Research

This study acknowledges several limitations that may affect the generalisability and comprehensiveness of the findings.

The sample size was relatively small, with only six participants interviewed out of approximately fifty potential respondents. While qualitative research often involves smaller sample sizes to allow for the in-depth exploration of experiences [11], the limited number of participants may not fully capture the diversity of perspectives within the broader population of responders.

Not all response agencies that were contacted responded to participate in the study. This non-participation could introduce bias, as the views of those who did not respond may differ from those who did. The agencies that did not participate might have had unique insights or faced different challenges that were not captured in this research.

This study focused exclusively on individuals who were physically present at the incident ground level during the Edgecumbe flood’s response phase. This exclusion criterion was implemented to maintain a manageable scope and focus the research on ground-level operations. However, it means that the perspectives of those working in the emergency operations centre (EOC) or other offsite locations were not included. These individuals may have had different experiences and insights that could have enriched this study’s findings.

Due to the constraints mentioned above, it is likely that not all considerations and issues related to the emergency response were identified. This study’s findings should therefore be interpreted with caution, and future research should aim to include a broader range of participants and perspectives to provide a more comprehensive understanding of the response to the Edgecumbe flood.

Further research is needed to explore the long-term impacts on both animals and their owners following a disaster. This includes studying the ongoing care requirements for rescued animals and the emotional and psychological effects on owners who were separated from their pets.

## 7. Conclusions

The 2017 Edgecumbe flood triggered the largest companion animal rescue operation in New Zealand’s history at the time, highlighting significant gaps in disaster preparedness for animals. This study explored the experiences of six first responders from various agencies involved in the rescue efforts, revealing several key challenges and areas for improvement in emergency response planning and execution.

The findings underscore the complex and multifaceted nature of animal rescue operations during disasters. Three main themes emerged from the analysis: the difficulties encountered during the initial rescue phase, the complex issues that arose after animals were rescued, and the health, safety, and welfare impacts on the human responders. These themes highlight the need for comprehensive planning and preparation to address both immediate and long-term challenges for animals, owners, and emergency personnel.

The key issues identified include the influence of the socio-zoological scale on rescue prioritisation, difficulties with feeding animals in place, the diversity of species requiring rescue, the importance of proper animal identification and tracking systems, the need for efficient animal decontamination procedures, and the emotional and logistical challenges of managing deceased animals. The long-term impacts on both animals and owners during the recovery phase underscore the necessity of comprehensive support services and collaborative efforts between animal welfare organisations, government agencies, and community groups.

This study also revealed pre-existing animal welfare issues and gaps in animal health infrastructure that were exposed and exacerbated by the disaster. This emphasises the importance of ongoing efforts to build resilience and preparedness in communities, not only for disaster response, but also for overall animal welfare.

Human factors, such as the health and mobility challenges faced by evacuees, the risks to responder safety, and the potential for responder fatigue during prolonged disaster operations, further highlight the need for holistic and integrated emergency response planning that addresses the interconnected needs of both humans and animals.

By learning from the experiences and insights of those involved in the Edgecumbe flood response and by incorporating these lessons into future policy, planning, and practice, we can enhance the effectiveness and compassion of emergency response efforts. This ultimately promotes the wellbeing and resilience of both human and animal communities in the face of disaster.

Future research should aim to include a broader range of participants and perspectives to provide a more comprehensive understanding of animal rescue operations in disasters. Additionally, studies exploring the long-term impacts on both animals and their owners following a disaster would be valuable in informing recovery planning and support services.

In conclusion, this study contributes to the growing body of knowledge on animal-inclusive emergency management and underscores the critical importance of incorporating animal welfare considerations into all aspects of disaster preparedness, response, and recovery. Continuous evaluation, improvement, and collaboration will be essential to ensure that the valuable lessons from Edgecumbe are not lost, but rather used to inform and strengthen our collective capacity to respond to future crises.

## Figures and Tables

**Table 1:** Timeline of response adapted from Rangitāiki River Scheme Review Panel, [2], Wellington SPCA National Rescue Unit after-action report [3], and transcripts.

Date (2017)	Time	Event
4 April		MetService issues first severe weather warning for area
5 April		Edgecumbe Schools are closed due to weather
5 April		Whakatane Emergency Operations Centre activated
6 April	0700–0730	Community start to self-evacuate township of Edgecumbe
6 April	0745–0800	Fire Service truck arrives (0751) at floodwall
6 April	0800–0815	Second Fire Service truck arrives, additional resources requested.
6 April	0800	Whakatane Emergency Response Team (NZRT17) activated by Whakatane District Council and deployed to Poroporo.
6 April	0830	Floodwall breaches and emergency evacuation undertaken by fire service and police. Whakatane Emergency Response Team diverted to Edgecumbe to assist with evacuation and arrive after most people evacuated, so focused on animals. All human occupants evacuated.
6 April	1240	Whakatane District Council declares state of emergency
6 April		SPCA National Rescue Unit and Wellington SPCA Emergency Reserve (emergency response assets) mobilised from Wellington and travel through the night to Whakatane.
7 April		SPCA emergency response assets report to Whakatane EOC in morning for briefing, then undertake initial reconnaissance of surrounding area of Edgecumbe and make entry into the cordoned township late afternoon with support from local SPCA Inspector and Whakatane Emergency Response Team (NZRT17). Additional NZRTs requested by SPCA to assist rescue efforts but declined by MPI coordinator.
8 April	Morning	Incident control point (ICP) set up outside Edgecumbe shops. Major animal rescue operation commences with SPCA, NZRTs (NZRT17 and NZRT15), and VERT with support from local fire brigade and other agencies. Animal rescue operation led by SPCA (as Incident Control) due to Fire Service Urban Search & Rescue declining to lead as no mandate for animal rescue. Rescue operation lasts for seven days.

## Data Availability

The original contributions presented in the study are included in the article; further inquiries can be directed to the corresponding author/s.
